# Retinal Microvascular Changes in Subtypes of Ischemic Stroke

**DOI:** 10.3389/fneur.2020.619554

**Published:** 2021-01-27

**Authors:** Ying Zhao, Bing Yang, An-Ding Xu, Yi-Wen Ruan, Ying Xu, Hui-Ling Hu, Ze-Feng Tan

**Affiliations:** ^1^Department of Neurology and Stroke Center, The First Affiliated Hospital, Jinan University, Guangzhou, China; ^2^Clinical Neuroscience Institute, The First Affiliated Hospital, Jinan University, Guangzhou, China; ^3^Department of Central Nervous System Regeneration, Guangdong-Hongkong-Macau Institute of Central Nervous System (CNS) Regeneration (GHMICR), Jinan University, Guangzhou, China; ^4^Shenzhen Key Laboratory of Ophthalmology, Shenzhen Eye Hospital, Shenzhen University School of Medicine, Shenzhen, China; ^5^Department of Neurology, The Affiliated Shunde Hospital of Jinan University, Guangzhou, China

**Keywords:** ischemic stroke, subtypes, retinal microvascular changes, venular diameter, neurolucida

## Abstract

**Aims:** Retinal microvasculature shares prominent similarities with the brain vasculature. We aimed to assess the association between retinal microvasculature and subtypes of ischemic stroke.

**Method:** We consecutively enrolled ischemic stroke patients within 7 days of onset, who met the criteria of subtype of atherothrombosis (AT), small artery disease (SAD), or cardioembolism (CE) according to a modified version of the Trial of Org 10172 in Acute Stroke Treatment (NEW-TOAST). Digital fundus photographs were taken within 72 h of hospital admission using a digital camera (Topcon TRC-50DX), and fundus photographs were semi-automatically measured by software (Canvus 14 and NeuroLucida) for retinal vasculature parameters.

**Results:** A total of 141 patients were enrolled, including 72 with AT, 54 with SAD, and 15 with CE. AT subtype patients had the widest mean venular diameter within 0.5–1.0 disk diameter (MVD_0.5−1.0DD_) followed by SAD and CE subtypes (86.37 ± 13.49 vs. 83.55 ± 11.54 vs. 77.90 ± 8.50, respectively, *P* = 0.047); CE subtype patients had the highest mean arteriovenous ratio within 0.5–1.0 disk diameter (MAVR_0.5−1.0DD_) followed by the AT and SAD subtype groups (0.97 ± 0.03 vs. 0.89 ± 0.99 vs. 0.89 ± 0.11, respectively, *P* = 0.010); SAD subtype patients were found with the highest mean venular tortuosity within 0.0–2.0 disk diameter (MVT_0.0−2.0DD_) followed by the AT and CE subtypes (1.0294 ± 0.0081 vs. 1.0259 ± 0.0084 vs. 1.0243 ± 0.0066, respectively, *P* = 0.024). After adjusting for clinic characteristics, MVD_0.5−1.0DD_ was significantly different among AT, SAD, and CE subtypes (*P* = 0.033). By receiver operating characteristic curve analysis, MVD_0.5−1.0DD_ predicted the AT subtype (area 0.690, 95% confidence interval, 0.566–0.815), with a cutoff value of 82.23 μm (sensitivity 61.1%, specificity 73.3%).

**Conclusion:** Retinal MVD_0.5−1.0DD_ (>82.23 μm) might be associated with the AT stroke subtype; however, we need large-scale prospective studies in future to explore the underlying mechanism and causal explanation for this finding.

## Introduction

The retinal microvasculature shares common features with the cerebral circulation (1). Studying the retinal microvasculature contributes to our understanding of pathological process of ischemic stroke. Evidence of retinal microvascular signs (such as microaneurysms, hemorrhages, arteriovenous nicking, and focal arteriolar narrowing) were associated with transient ischemic attack (TIA) as well as stroke (2–4) and could predict progression of brain microvascular diseases (leukoaraiosis and lacunar infarcts) (5). Studies had shown that variation in retinal arteriolar diameter was associated with intracranial arterial stenosis (6), and larger retinal venular diameters were associated with an increased risk of stroke (7).

These findings further supported that retinal microvascular abnormalities were associated with stroke, but more data were required to clarify associations between specific types of retinal microvascular abnormality and subtypes of stroke (8). A few studies had investigated the relationship between retinal vascular parameters and subtypes of ischemic stroke with inconsistent conclusions. Doubal et al. (9) found that patients with lacunar infarction had wider venular diameter and narrower arterial diameter than patients with cortex infarction. Some other studies also suggested that retinal vascular abnormalities (narrower arterial diameter, wider venular diameter, arteriovenous nicking, and focal arteriolar narrowing) were associated with lacunar infarction (10–13). In contrast, Ong et al. (14) found no difference in retinal vascular parameters between stroke subtypes using a modified version of the Trial of Org 10172 in Acute Stroke Treatment (NEW-TOAST) classification. The different methods of stroke classification in previous studies may be one of the reasons for the inconsistent results.

The NEW-TOAST classification is the most widely used and approved form of etiologic subtyping with high inter-rater reliability (kappa = 0.82) (15). Clinical therapies and secondary prevention strategies vary among stroke subtypes and have significant impacts on patient outcomes (16).

However, stroke subtyping relies on detailed history taking and complementary investigations, which are not conducive to screening. This study aimed to assess the association between retinal microvascular parameters and subtypes of ischemic stroke defined by NEW-TOAST classification.

## Methods

### Study Subjects

**Figure 1:**
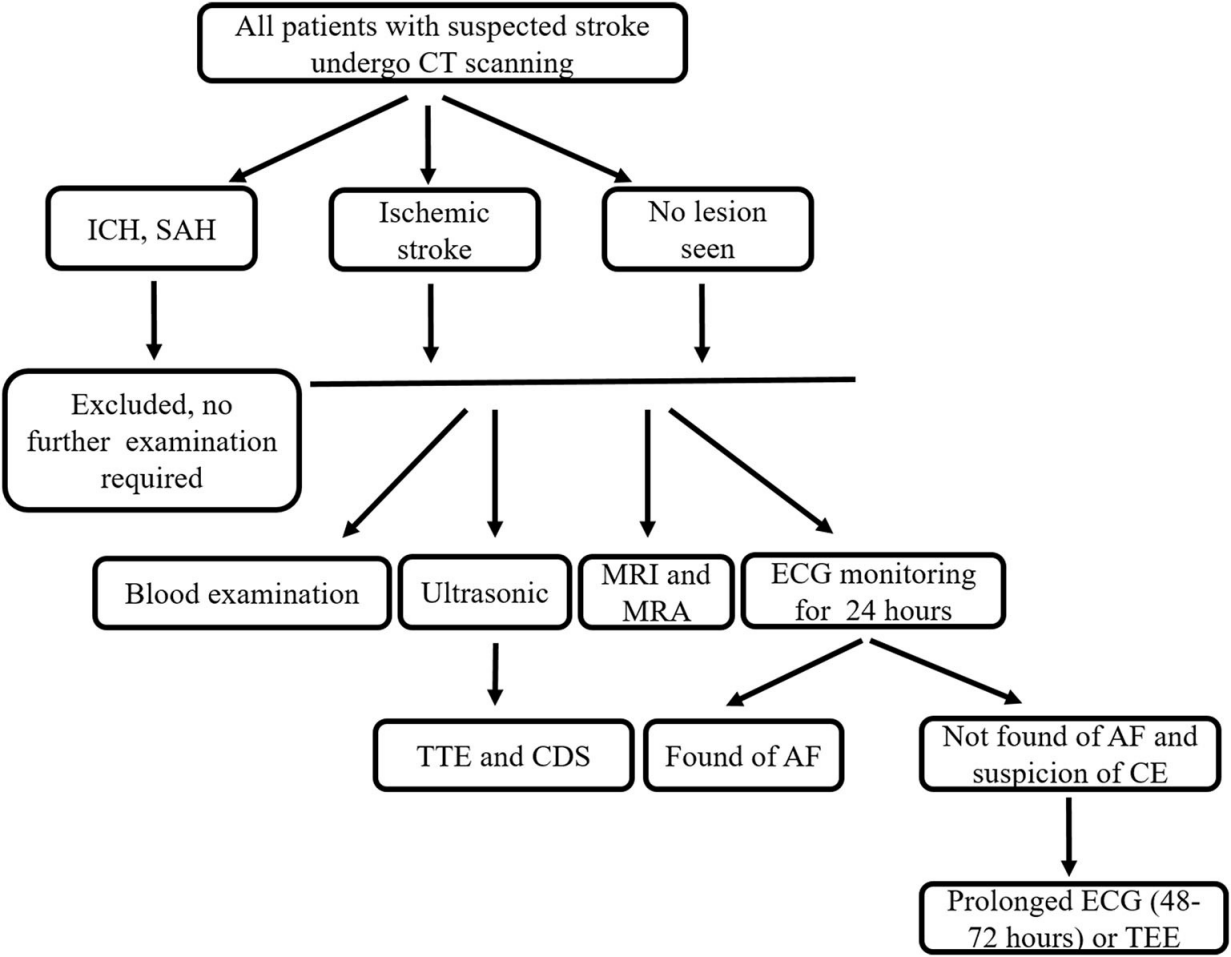
Examination flow chart. CT, computer tomography; ICH, intracerebral hemorrhage; SAH, subarachnoid hemorrhage; MRI, magnetic resonance imaging; MRA, magnetic resonance angiography; ECG, electrocardiogram; TTE, transthoracic echocardiography; CDS, carotid duplex sonography; AF, atrial fibrillation; CE, cardioembolism; TEE, transesophageal echocardiography.

From October 2015 to March 2017, we consecutively included patients with ischemic stroke admitted to our stroke center within 7 days of onset. We recorded patient demographics, vascular risk factors, past medical history, neurological deficit, the National Institutes of Health Stroke Scale (NIHSS) scores, and acute vascular recanalization therapies after admission, as well as results of stroke-related investigation (detailed information in examination flow chart, Figure 1). We determined the patient's stroke subtype according to the NEW-TOAST criteria based on the patient's clinical findings. We included all patients over 18 years with New-TOAST stroke subtypes of atherothrombosis (AT), small artery disease (SAD), or cardioembolism (CE) and excluded patients who had not undergone fundus photograph examination or with ocular diseases that affected the visualization of fundus photography.

### Assessment of Retinal Vascular Parameters

Digital fundus photographs were taken within 72 h of hospital admission by dilating with 1% tropicamide eye drops as standardized protocol. We used a camera (Topcon TRC-50DX) to photograph bilateral retinal vasculature. Photographs were centered on the region of the optic disc and were taken in 50° range. We took the average of each retinal vasculature parameter values on both sides as the final values. We drew a scaleplate in the fundus camera to get the actual size of diameter and measured the scaleplate in NeuroLucida program.

**Figure 2:**
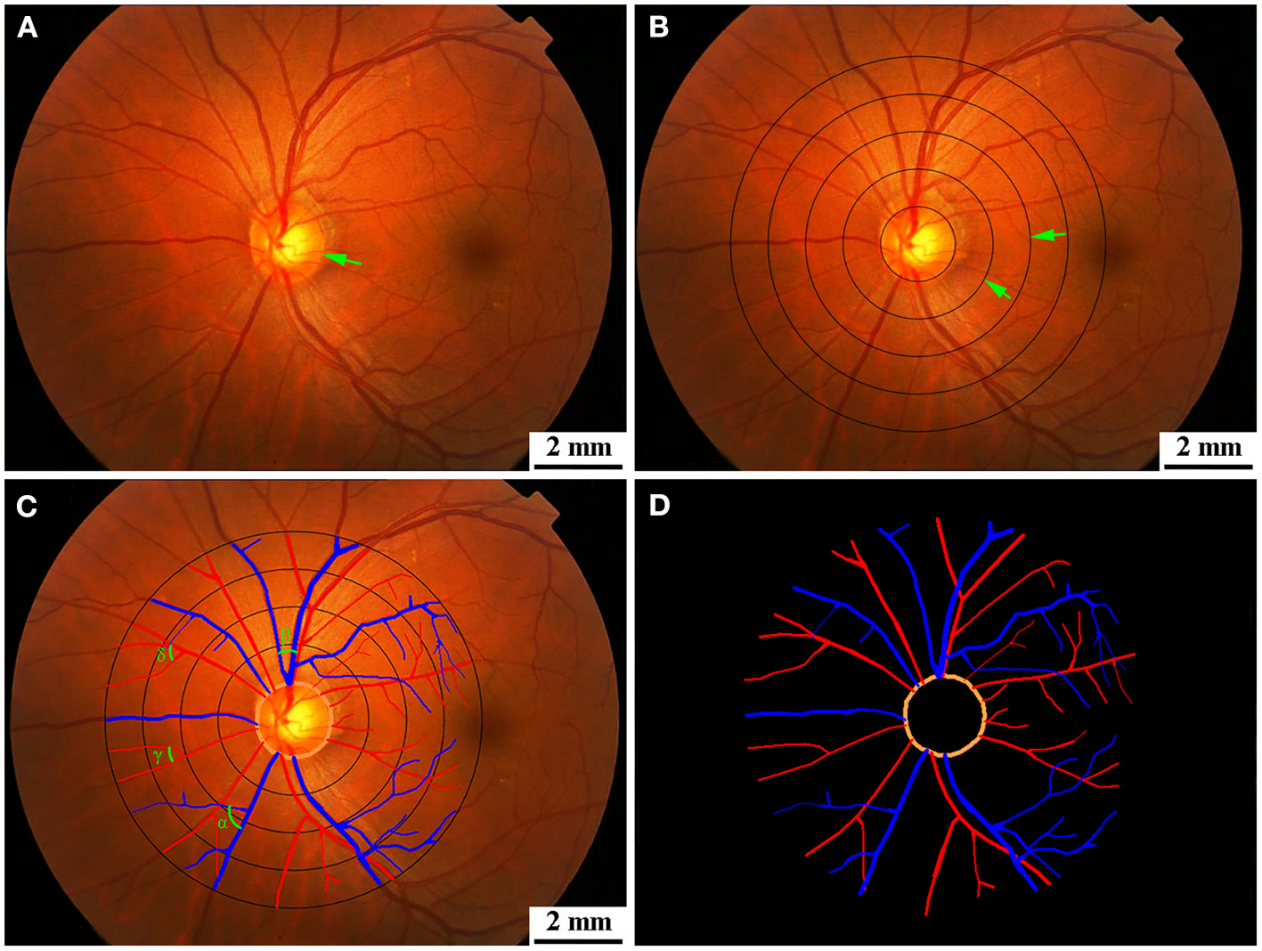
**(A)** Original diagram. The green arrow showed optic disk. **(B)** Retinal photographs graded by Canvus 14, drew concentric circles within the scope of 0.0–2.0 DD from the optic disk margin per interval distance 0.5 DD. The green arrows showed concentric circles. **(C)** Retinal photographs graded by NeuroLucida. Arteries are traced as red line, and veins are traced as blue line. The branching angles of vessels are shown as α, β, γ, and δ in **(C)**. **(D)** Segmentation of retina vessels. DD, disk diameter.

All fundus photographs were graded at the Guangdong-Hongkong-Macau Institute of Central Nervous System (CNS) Regeneration (GHMICR) of Jinan University using software Canvus 14 (Advanced Cell Diagnostics, Inc.) and NeuroLucida (MicroBrightField, Inc.). Trained graders, without knowledge of the identification of participants, used the Canvus 14 to draw concentric circles within the scope of 0.0–2.0 disc diameters (DD) from the optic disk margin per interval distance 0.5 DD (Figures 2A,B). Vessels were traced according to the diameter and trajectory by software NeuroLucida (Figure 2C). Prior to tracing the vessels, we labeled arteries and veins according to their different characteristics (thin and pink for arteries, thick and purple for veins). For better observation of the morphological characteristic of target vessels, we extracted vascular images from the background of fundus (Figure 2D). Software Neurolucida automatically calculated the diameter, tortuosity, and branching angle of the vessels. We measured retinal vascular parameters (vascular diameter, arteriovenous ratio, tortuosity, and branching angle) and analyzed them.

### Definition and Automatic Calculation of Retinal Vascular Parameters

The DD was measured by drawing a circle along the optic disk margin, which was calculated by software NeuroLucida. Retinal vascular diameters (the internal size of the vessel) were calculated as the average diameter of arteries or veins coursing from 0.5 to 1.0 DD by software NeuroLucida and expressed as mean arterial diameter (MAD) or mean venular diameter (MVD). The mean arteriovenous ratio (MAVR) from 0.5 to 1.0 DD was defined as the ratio of MAD and MVD (MAD/MVD). Retinal vascular tortuosity was defined as the integral of the curvature square along the vessel path, normalized by the total path length (17). The tortuosity of retinal arteries or veins from 0.0 to 2.0 DD were expressed as mean arterial tortuosity (MAT) or mean venular tortuosity (MVT) and automatically calculated by software NeuroLucida. Smaller values of MAT or MVT represent less tortuosity of the retinal vessels. The retinal vascular branching angle was the first angle subtended between two daughter vessels at each vascular bifurcation (18). In this study, the average branching angle of the arteries and veins in 0.0–2.0 DD were automatically calculated by software NeuroLucida and expressed as mean arterial branching angle (MABA) and mean venular branching angle (MVBA), respectively.

### Assessment of Ischemic Stroke Subtypes

The classification of stroke subtype was based on the results of routine hospital investigations including blood examination, transthoracic echocardiography (TTE), carotid duplex sonography (CDS), 24 h electrocardiogram (ECG) monitoring and brain imaging [computer tomography (CT), magnetic resonance imaging (MRI), and MR angiography (MRA)] (see examination flow chart, Figure 1). In order to screen patients for CE, patients with clinical suspicion of CE underwent prolonged ECG (48–72 h) or transesophageal echocardiography (TEE) according to our routine hospital protocol. Two senior neurologists with over 10 years of experience (Z.T. and Y.Z.) classified the different stroke subtypes according to the NEW-TOAST classification (15, 19). Discrepancies between the two evaluators were decided after consultation with another neurologist A.X. (more than 15 years of experience). The specific standards and methods of classification were performed as described previously (15).

### Statistical Analysis

All data were analyzed with the statistical software program IBM SPSS Statistics 25. One-way analysis of variance (ANOVA) followed by the Student–Newman–Keuls (SNK) multiple comparison tests was used for continuous variables. Pearson chi-square test comparing multiple sample rates was used for categorical variables. We used one-way ANOVA and Pearson chi-square test for assessing the differences in retinal vascular parameters among different subtypes of ischemic stroke. The analysis of covariance was used for adjusting demographic and clinic characteristics [age, sex, current cigarette smoker, hypertension, diabetes, dyslipidemia, stroke history, coronary disease, peripheral artery disease, and body mass index (BMI)]. We applied receiver operating characteristic (ROC) curve to evaluate the diagnostic value of MVD_0.5−1.0DD_ for AT stroke subtype. *P* < 0.05 were considered as significant.

## Results

**Figure 3:**
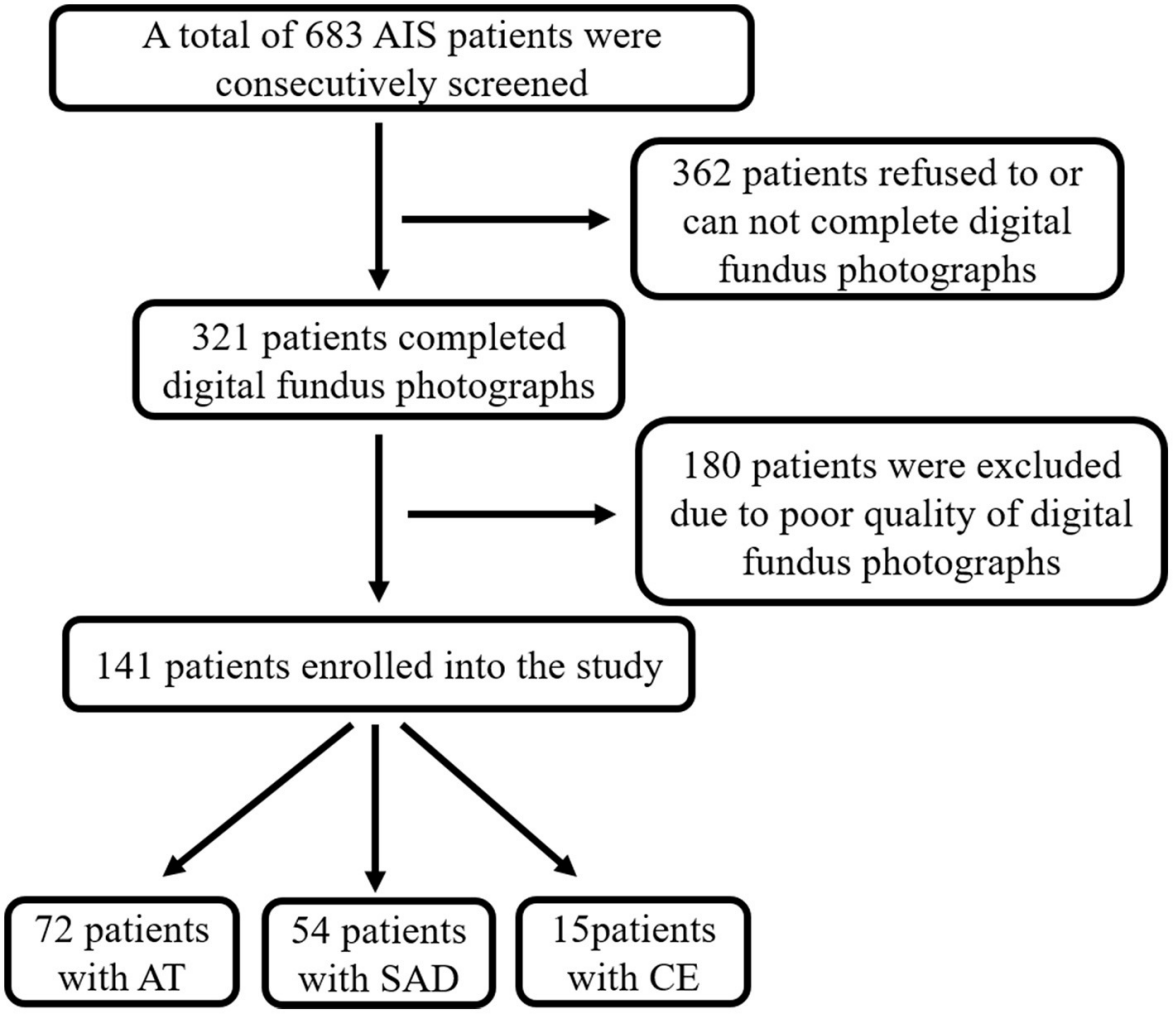
Screening flow chart. AIS, acute ischemic stroke; AT, atherothrombosis; SAD, small artery disease; CE, cardioembolism.

From October 2015 to March 2017, 141 acute ischemic stroke (AIS) patients were enrolled in this study with qualified retinal microvascular images (see screening flow chart, Figure 3). Among them, 72 patients were AT, 54 patients belonged to SAD, and 15 were geared to CE. The demographic and clinic characteristics of the subjects are shown in Table 1. No significant differences were found among AT, SAD, and CE subtype in gender, current smoking, diabetes, stroke history, coronary disease, peripheral artery disease, BMI, Modified Rankin Scale (mRS), Barthel Index (BI), and NIHSS. Among three subtype groups, the SAD subtype was the youngest (*P* = 0.001). The CE subtype had the least proportion of hypertension (*P* = 0.009) and hyperlipidemia (*P* = 0.000) and the highest rate of acute recanalization treatment (*P* = 0.005).

**Table 1:** Comparison of demographic and clinic characteristics among AT, SAD, and CE subtypes.

	**AT**	**SAD**	**CE**	***P***
*n*	72	54	15	—
Age (years), mean (SD)	62.58 (8.94)	55.61 (10.97)	61.73 (15.45)	0.001^*^
Male, *n* (%)	46 (63.8)	42 (77.8)	9 (60.0)	0.552
Current cigarette smoker, *n* (%)	27 (37.5)	23 (2.6)	2 (13.3)	0.114
Hypertension, *n* (%)	63 (87.5)	43 (79.6)	8 (53.3)	0.009^*^
Diabetes, *n* (%)	23 (31.9)	18 (33.3)	1 (6.7)	0.115
Hyperlipidemia, *n* (%)	42 (58.3)	36 (66.7)	1 (6.7)	0.000^*^
Stroke history, *n* (%)	15 (20.8)	14 (25.9)	3 (20.0)	0.769
Coronary disease, *n* (%)	7 (9.7)	1 (1.9)	1 (6.7)	0.202
Peripheral artery disease, *n* (%)	0 (0.0)	0 (0.0)	1 (6.7)	0.103
Acute recanalization treatment, *n* (%)	1 (1.4)	0 (0.0)	2 (13.3)	0.005^*^
BMI of 30 or more, *n* (%)	2 (2.8)	3 (5.6)	0 (0.0)	0.519
mRS, mean (SD)	2.13 (1.26)	1.18 (1.16)	1.87 (1.36)	0.683
BI, mean (SD)	75.69 (26.50)	80.00 (20.35)	77.00 (28.34)	0.621
NIHSS, mean (SD)	3.83 (2.77)	3.43 (2.30)	3.27 (3.67)	0.615

*AT, atherothrombosis; SAD, small artery disease; CE, cardioembolism; BMI, body mass index; mRS, Modified Rankin Scale; BI, Barthel Index; NIHSS, National Institutes of Health Stroke Scale*.

* *P < 0.05*.

**Figure 4:**
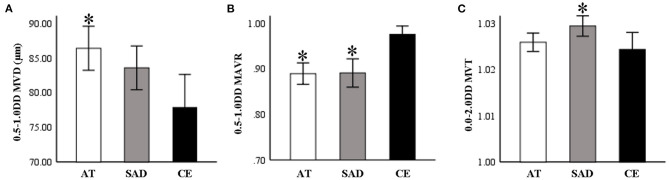
**(A)** AT subtype group had the widest MVD_0.5−1.0DD_ (*P* = 0.047). **(B)** CE subtype group had the highest MAVR_0.5−1.0DD_ (*P* = 0.010). **(C)** The SAD subtype group had the largest MVT_0.0−2.0DD_ (*P* = 0.024). AT, atherothrombosis; SAD, small artery disease; CE, cardioembolism; DD, disk diameter; MVD, mean venular diameter; MAVR, mean arteriovenous ratio; MVT, mean venular tortuosity. *P < 0.05, compared to CE.

As shown in Table 2, AT subtype had the widest MVD_0.5−1.0DD_ (*P* = 0.047, Figure 4A), CE subtype had the highest MAVR_0.5−1.0DD_ (*P* = 0.010, Figure 4B), and the SAD subtype had the largest MVT_0.0−2.0DD_ (*P* = 0.024, Figure 4C).

**Table 2:** Comparison of retinal vascular parameters among AT, SAD, and CE subtypes.

	**AT**	**SAD**	**CE**	***P***
*n*	72	54	15	—
DD (μm), mean (SD)	1,794.53 (283.41)	1,808.38 (293.03)	1,832.79 (125.48)	0.877
MAD_0.5−1.0DD_ (μm), mean (SD)	75.35 (13.87)	73.88 (10.65)	75.96 (8.99)	0.749
MVD_0.5−1.0DD_ (μm), mean (SD)	86.37 (13.49)	83.55 (11.54)	77.90 (8.50)	0.047^*^
MAVR_0.5−1.0DD_, mean (SD)	0.89 (0.99)	0.89 (0.11)	0.97 (0.03)	0.010^*^
MAT_0.0−2.0DD_, mean (SD)	1.0042 (0.1212)	1.0186 (0.0085)	1.0196 (0.0075)	0.607
MVT_0.0−2.0DD_, mean (SD)	1.0259 (0.0084)	1.0294 (0.0081)	1.0243 (0.0066)	0.024^*^
MABA_0.0−2.0DD_ (°), mean (SD)	30.61 (5.68)	29.98 (3.67)	30.25 (3.50)	0.769
MVBA_0.0−2.0DD_ (°), mean (SD)	33.17 (3.89)	34.34 (3.40)	34.07 (4.96)	0.224

*AT, atherothrombosis; SAD, small artery disease; CE, cardioembolism; DD, disk diameter; MAD, mean arterial diameter; MVD, mean venular diameter; MAVR, mean arteriovenous ratio; MAT, mean arterial tortuosity; MVT, mean venular tortuosity; MABA, mean arterial branching angle; MVBA, mean venular branching angle*.

* *P < 0.05*.

**Figure 5:**
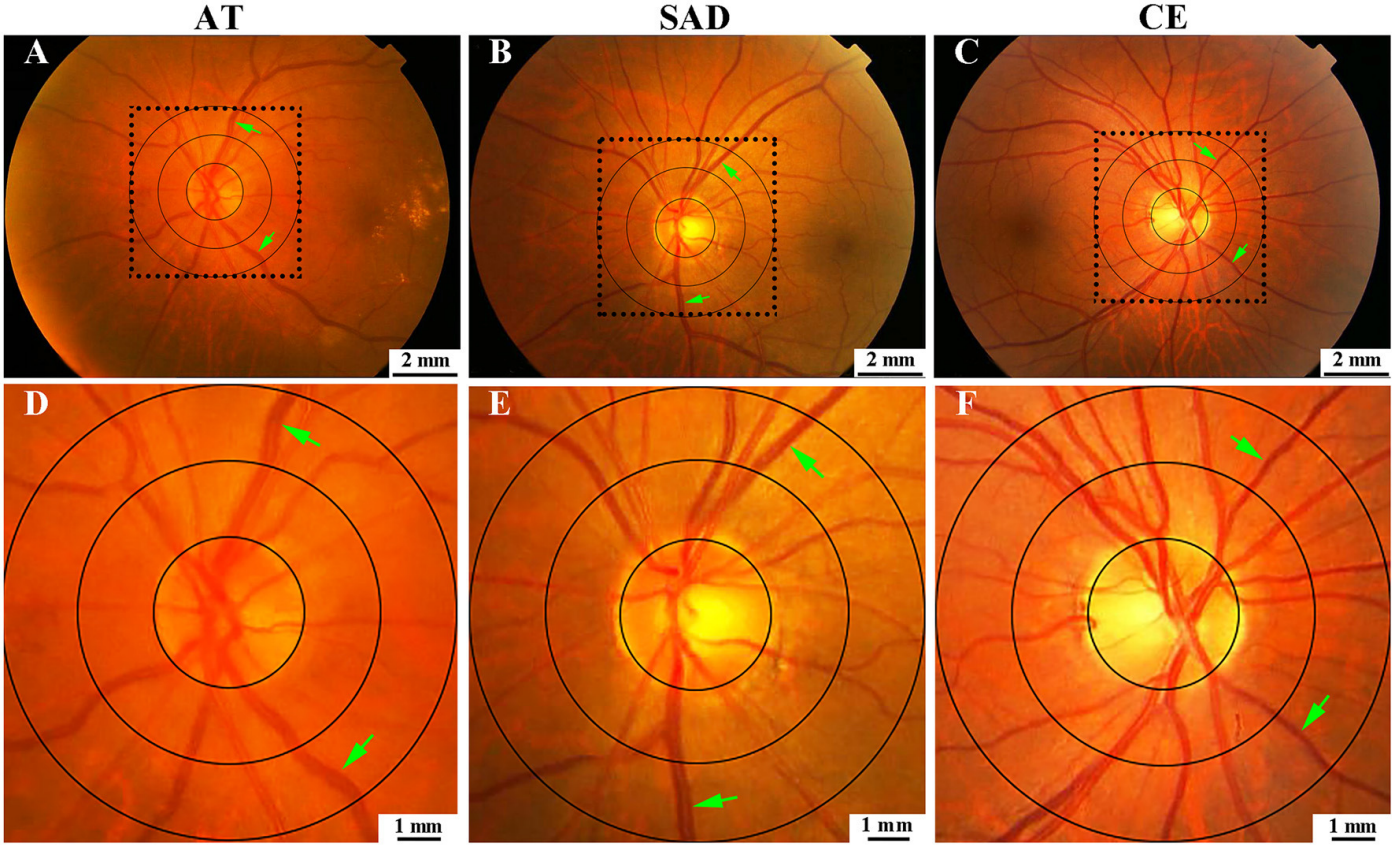
Comparison of MVD_0.5−1.0DD_ among AT, SAD, and CE subtypes. **(A)** was for AT, **(B)** was for SAD, **(C)** was for CE. **(D–F)** were the magnified images of the selected areas in **(A–C)**, respectively. AT, atherothrombosis; SAD, small artery disease; CE, cardioembolism; DD, disk diameter; MVD, mean venular diameter.

Comparison of MVD_0.5−1.0DD_, MAVR_0.5−1.0DD_, and MVT_0.0−2.0DD_ among three subtypes is shown in Table 3 after adjusting for age, sex, current cigarette smoker, hypertension, diabetes, dyslipidemia, stroke history, coronary disease, peripheral artery disease, and BMI. MVD_0.5−1.0DD_ was significantly different among the three stroke subtypes (Figure 5). Nevertheless, there were no differences in MAVR_0.5−1.0DD_ and MVT_0.0−2.0DD_ after adjusting for clinic characteristics.

**Table 3:** Comparison of MVD_0.5−1.0DD_, MAVR_0.5−1.0DD_, and MVT_0.0−2.0DD_ among AT, SAD, and CE subtypes after adjusting for clinic characteristics.

	**AT**	**SAD**	**CE**	***P***
*n*	72	54	15	—
MVD_0.5−1.0DD_ (μm)	86.37 (13.49)	83.55 (11.54)	77.90 (8.50)	0.000^*^
MAVR_0.5−1.0DD_	0.89 (0.99)	0.89 (0.11)	0.97 (0.03)	0.105
MVT_0.0−2.0DD_	1.0259 (0.0084)	1.0294 (0.0081)	1.0243 (0.0066)	0.129

*Adjusted for age, sex, current cigarette smoker, hypertension, diabetes, dyslipidemia, stroke history, coronary disease, peripheral artery disease, and BMI. AT, atherothrombosis; SAD, small artery disease; CE, cardioembolism; DD, disk diameters; MVD, mean venular diameter; MAVR, mean arteriovenous ratio; MVT, mean venular tortuosity; BMI, body mass index*.

* *P < 0.05*.

**Figure 6:**
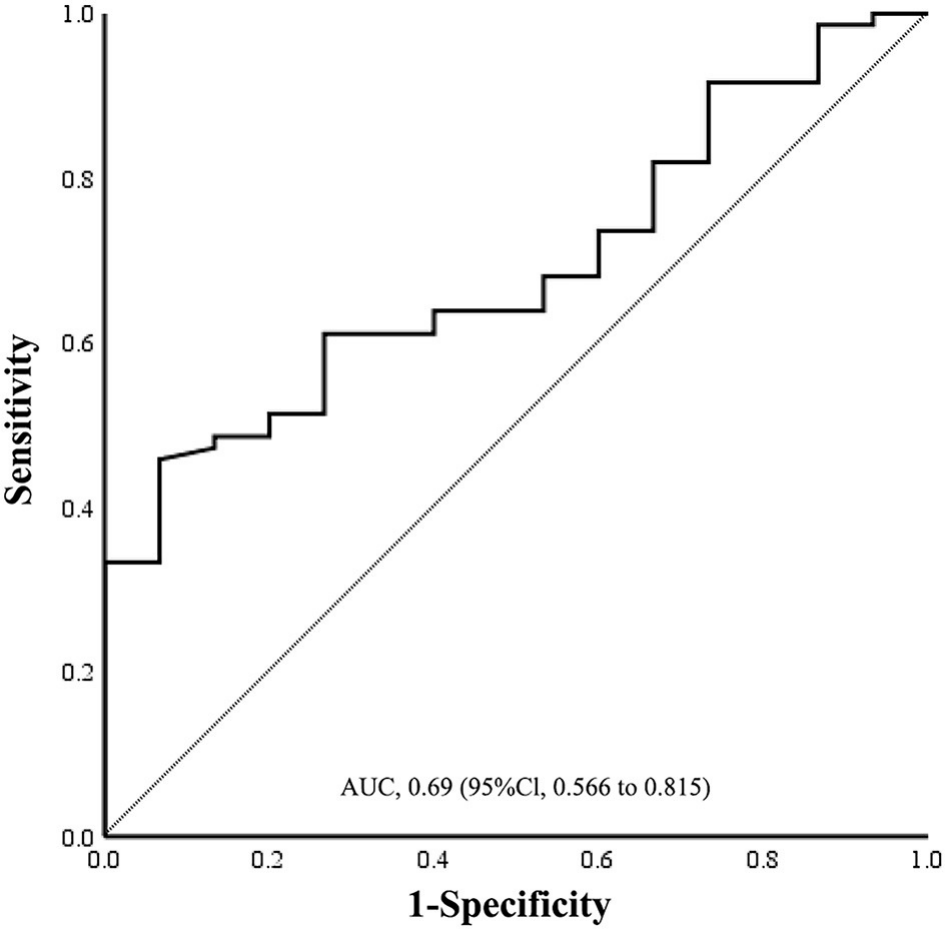
Sensitivity and specificity of ROC curve in the assessment of prediction of AT by MVD_0.5−1.0DD_. Cutoff value was 82.23 μm. Sensitivity was 61.1%, and specificity was 73.3%. ROC, receiver operating characteristic; AUC, area under curve; AT, atherothrombosis; MVD, mean venular diameter.

The ROC curve was used to evaluate the diagnostic value of MVD_0.5−1.0DD_ between subtype group AT and CE. As shown in Figure 6, area under ROC curve (AUC) for prediction of AT subtype by MVD_0.5−1.0DD_ was 0.690 [95% confidence interval (CI): 0.566–0.815, *P* = 0.021]. The cutoff value of MVD_0.5−1.0DD_ for the AT subtype classification was 82.23 μm with a sensitivity of 61.1% and specificity of 73.3%.

## Discussion

In this study, we found significant differences in MVD_0.5−1.0DD_ among different stroke subtypes. AT subtype had the widest MVD_0.5−1.0DD_, and MVD_0.5−1.0DD_ (>82.23 μm) was associated with the classification of AT stroke subtype.

Previous research has shown that retinal vessel diameter was associated with stroke risk (8, 20). A meta-analysis showed that wider retinal venular diameter predicted stroke (pooled hazard ratio = 1.15, 95% CI: 1.05–1.25 per 20 μm increase in diameter) but not the diameter of retinal arterioles (20). Similar results were reported in another systematic review that wider retinal venular diameter increased the risk of incident stroke by 40% (summary risk ratios = 1.4, 95% CI: 1.1–1.7) (8). These provided shreds of evidence that wider retinal venular diameter was associated with stroke. The mechanisms linking wider retinal venules and stroke were unclear. Concurrent circulatory phenomena in both the retina and brain, such as altered vessel wall stresses, cerebral hypoxia, or venous insufficiency might be the underlying causes (21, 22). Other studies suggested that a wider retinal venular caliber was a marker of endothelial dysfunction and inflammation, as well as an indicator of impaired cerebral oxygen perfusion (23, 24).

Our study found that AT subtype was associated with wider venular diameter. However, we still lack an explanation of the mechanism and causal relationship in this phenomenon. There were few studies that investigated the relationship between retinal vascular parameters and subtypes of ischemic stroke. Consistent with our study, Ikram et al. (25) found an association between atherosclerosis and larger venular diameters. The present study was one of the few studies that found the association between AT subtype and retinal venular diameter in the Asian population.

One of the possible underlying mechanisms of the association between retinal venules and AT subtype may be due to their sharing risk factors for atherosclerosis. Wider retinal venules were more common in diabetic retinopathy (26), and diabetes mellitus was a risk factor for atherosclerosis. Patients with retinal venous thrombosis or deep venous thrombosis were more often associated with atherosclerosis (27, 28). Both atherosclerosis and venous thrombosis were linked to similar risk factors, such as hyperlipidemia, platelet activation, and blood coagulation (28). However, this trend was found controversial in other studies. One study showed that lacunar stroke subtype was associated with wider retinal venular caliber than other stroke subtypes (10, 11), and some other studies showed there were no significant differences in retinal vascular parameters among stroke subtypes (14, 29). One of the reasons for the inconsistency may be the different methods of stroke classification used in different studies.

Software Neurolucida was initially used to perform morphometric measurements of neurons and dendrites (30). The neuron bodies morphologically resemble the optic disc, and the axons and dendrites resemble the retinal vasculature; therefore, we used software Neurolucida for the first time to track the retinal vascular morphology and obtain retinal vascular parameters. We used software to measure all target vessels' actual mean values within the detection range, rather than to measure the relative mean values of some selected vessels as in previous studies. The accuracy and consistency of the software Neurolucida had been demonstrated by the DD we obtained being consistent with the actual diameter in previous studies (9, 31). Similar to our study, some studies used the other computer-assisted program for the measurement of retinal vascular parameters (such as computer monitor or computer imaging program of retinal analysis) (11, 32, 33). In a few studies, retinal photographs were graded by semi-automated computer software, Singapore I Vessel Assessment (SIVA). By selecting the diameters of a main vessel and two branch vessels, the central retinal arteriolar equivalent (CRAE) and central retinal venular equivalent (CRVE) were calculated as the revised Knudtson–Parr–Hubbard formula (14, 34), and selection bias was unavoidable by this approach. This study measured the average diameter of all arterioles and venules coursing through an area of 0.5–1.0 DD from the optic disc margin, which provides us a global reflection of the retinal micro-circulation.

There were some limitations to the present study. Firstly, the present study had a relatively small sample size. Second, the proportion of CE subtype in the present study was relatively lower than other studies, even though most of the CE subtypes can be identified by routine examination. Patients with massive infarction or in serious condition were excluded due to incompetent to complete digital fundus photography examination, especially CE subtype patients with large vessel occlusion. So the present study was not a full reflection of stroke subtypes in the Asian population. Thirdly, we did not set healthy people as control group in this study. In this study, we mainly focused on the difference of retinal vascular parameters in various ischemic stroke subtypes, rather than comparison between healthy and ischemic stroke patients. Furthermore, previous studies also suggested that retinal vascular parameters were associated with ischemic stroke after adjusting for age, sex, race, and cerebrovascular risk factors (2, 8, 14, 35). At last, stroke of other demonstrated etiology (SOE) and stroke of other undemonstrated etiology (SUE) were not included in this study because of their unclear causes of stroke. We could not rule out the possibility that part of them might be AT, CE, or SAD subtypes. Therefore, we looked forward to large-scale longitudinal cohort study focused on observing the association between retinal microvascular changes and followed up ischemic stroke subtype in the near future.

## Conclusion

Retinal MVD_0.5−1.0DD_ (>82.23 μm) might be associated with the AT stroke subtype; however, we need large-scale prospective studies in future to explore the underlying mechanism and causal explanation for this finding.

## Data Availability Statement

The raw data supporting the conclusions of this article will be made available by the authors, without undue reservation.

## Ethics Statement

The studies involving human participants were reviewed and approved by The ethics committees of the First Affiliated Hospital of Jinan University. The patients/participants provided their written informed consent to participate in this study. Written informed consent was obtained from the individual(s) for the publication of any potentially identifiable images or data included in this article.

## Author Contributions

Z-FT and H-LH conceived and designed the study. YZ, BY, Y-WR, and YX performed the study. YZ and BY analyzed the data. YZ, BY, Z-FT, and H-LH wrote the paper. A-DX gave suggestions how to design the study, edit the results, and write the manuscript. All authors contributed to the article and approved the submitted version.

## Conflict of Interest

The authors declare that the research was conducted in the absence of any commercial or financial relationships that could be construed as a potential conflict of interest.
